# Effect of socio-economic deprivation on refugee health: a natural experiment study in Germany

**DOI:** 10.1093/eurpub/ckac130.080

**Published:** 2022-10-25

**Authors:** L Biddle, K Bozorgmehr

**Affiliations:** 1 Population Medicine and Health Services Research, University Bielefeld, Bielefeld, Germany; 2 Section Health Equity Studies & Migration, University Hospital Heidelberg, Heidelberg, Germany

## Abstract

**Background:**

Characteristics of the place of residence have been proposed as a key determinant of physical and mental health, but so far, little experimental evidence exists. The quasi-random dispersal of refugees in Germany serves as a natural experiment to study the causal relationship between socio-economic deprivation and health as well as the impact of the social context on this relationship.

**Methods:**

Refugees subject to dispersal policy (n = 1723) were selected from the nation-wide German IAB-SOEP-BAMF Panel from 2016 to 2018. The effect of German Index of Socioeconomic Deprivation quintiles (Q1-Q5) on change between baseline (t0) and follow-up (t1) in mental (mcs) and physical (pcs) health component scales of SF-12 were analysed using multi-level linear regression. Social context variables were included in a mediation analysis.

**Results:**

Across quintiles, mental health improves (Δmcs=0.5) and physical health declines (Δpcs=-0.8) between t0 and t1. Fully adjusted models show a negative, dose-responsive relationship between deprivation and physical health, which is statistically significant for Q4 (coef. Q4vsQ1: -1.84, 95%CI: -3.50;-0.17). Models for mental health show an improvement in Q5 (coef. Q5vsQ1: 6.00, 95%CI: 1.70;10.31). Social context variables have no effect on physical health but slightly diminish the effect on mental health (coef. Q5vsQ1: 4.78, 95%CI: 0.65;8.90).

**Conclusions:**

The quasi-random dispersal of refugees in Germany acts as a natural experiment to disentangle selection effects from the relationship between deprivation and health. Results suggest a negative effect of deprivation on physical health and a potential positive effect on mental health which can be partially explained by the social context. Limitations are the small sample sizes in deprived quintiles and short follow-up periods. This analysis can act as a magnifying glass for similar effects among other population groups, but causal paths need to be investigated further.

**Key messages:**

- The quasi-random dispersal of refugees in Germany acts as a natural experiment to disentangle selection effects from the relationship between deprivation and health.
- Results from a natural experiment suggest a negative effect of deprivation on physical health and a potential positive effect on mental health which can be partially explained by the social context.

